# *Himatanthus bracteatus*-Composed In Situ Polymerizable Hydrogel for Wound Healing

**DOI:** 10.3390/ijms232315176

**Published:** 2022-12-02

**Authors:** Bernadeth M. de Almeida, Izabella D. Dorta dos Santos, Felipe M. A. de Carvalho, Luana C. Correa, John L. S. Cunha, Claudio Dariva, Patricia Severino, Juliana C. Cardoso, Eliana B. Souto, Ricardo L. C. de Albuquerque-Júnior

**Affiliations:** 1 Biotechnological Postgraduate Program—RENORBIO, Tiradentes University, Aracaju 49010-390, SE, Brazil; 2 Postgraduate Program in Health and Environment, Tiradentes University, Aracaju 49032-490, SE, Brazil; 3 School of Physiotherapy, Tiradentes University, Aracaju 49032-490, SE, Brazil; 4 Department of Odontology, Paraiba State University, Campina Grande 58429 500, PB, Brazil; 5 Laboratory for Colloidal Systems Studies, Institute of Technology and Research (ITP), Tiradentes University, Aracaju 49010-390, SE, Brazil; 6 Department of Pharmaceutical Technology, Faculty of Pharmacy, University of Porto, Rua de Jorge Viterbo Ferreira, nº. 228, 4050-313 Porto, Portugal; 7 REQUIMTE/UCIBIO, Faculty of Pharmacy, University of Porto, Rua de Jorge Viterbo Ferreira, nº. 228, 4050-313 Porto, Portugal; 8 Department of Pathology, Federal University of Santa Catarina, Florianópolis 88040-370, SC, Brazil

**Keywords:** *Himatanthus bracteatus* ethanolic extract, gelatin methacryloyl hydrogel, GelMA, iridoids, Wistar rats

## Abstract

The *Himatanthus* genus presents anti-inflammatory, antioxidant activities, suggesting potential wound-healing properties. This study aimed to develop and analyze the wound-healing properties of a photopolymerizable gelatin-based hydrogel (GelMA) containing an ethanolic extract of *Himatanthus bracteatus* in a murine model. The extract was obtained under high pressure conditions, incorporated (2%) into the GelMA (GelMA-HB), and physically characterized. The anti-inflammatory activity of the extract was assessed using a carrageenan-induced pleurisy model and the GelMA-HB scarring properties in a wound-healing assay. The extract reduced IL-1β and TNF-α levels (48.5 ± 6.7 and 64.1 ± 4.9 pg/mL) compared to the vehicle (94.4 ± 2.3 pg/mL and 106.3 ± 5.7 pg/mL; *p* < 0.001). GelMA-HB depicted significantly lower swelling and increased resistance to mechanical compression compared to GelMA (*p* < 0.05). GelMA-HB accelerated wound closure over the time course of the experiment (*p* < 0.05) and promoted a significantly greater peak of myofibroblast differentiation (36.1 ± 6.6 cells) and microvascular density (23.1 ± 0.7 microvessels) on day 7 in comparison to GelMA (31.9 ± 5.3 cells and 20.2 ± 0.6 microvessels) and the control (25.8 ± 4.6 cells and 17.5 ± 0.5 microvessels) (*p* < 0.05). In conclusion, GelMA-HB improved wound healing in rodents, probably by modulating the inflammatory response and myofibroblastic and microvascular differentiation.

## 1. Introduction

Wound healing is a complex pathophysiological process that occurs to restore dermo-epidermal integrity after injury [1]. In this process, a variety of cell types with distinct roles act synchronously and asynchronously in the phases of hemostasis, inflammation, neovascularization, fibroplasia, re-epithelialization, and healing remodeling [2]. Changes that disrupt any of these steps, such as infections, underlying diseases, and medications, may impair and prolong the healing process [3].

The formation of new blood vessels (angiogenesis) is a critical step for the development of the granulation tissue that occurs in response to the release of growth factors such as vascular endothelial growth factor (VEGF) and platelet-derived growth factor (PDGF) [4]. During angiogenesis, endothelial cells found on the inner surface of blood vessels proliferate to form a network of capillary blood vessels that provides the supply of nutrients and oxygen needed for an efficient wound healing [5]. The proper modulation of angiogenesis has been considered a promising strategy to improve wound healing [6].

Another important step of the healing process is the wound contraction that occurs after the gradual collagenization of granulation tissue to form the primary fibrous scar. TGF-β is the main cytokine associated with the differentiation of fibroblasts into myofibroblasts [7], cells with a contractile phenotype, essential for reducing the wound area, but also responsible for connective tissue fibrosis [8]. The healing process is strongly dependent on the proper development of these two pathophysiological events, so that disturbances during these biological phenomena determine the formation of chronic wounds or misshapen scars [9].

Several studies on the healing potential of plant-derived natural products have been carried out [10]. The identified medicinal properties are related to the content of bioactive phytochemical constituents in plant extracts, such as alkaloids, iridoids, flavonoids, tannins, saponins, and phenolic compounds [11]. The main advantage of the use of plant extracts as healing agents is the complex combinations of phytoconstituents, allowing that the same product can act on different pathophysiological steps of wound healing, such as angiogenesis, fibroplasia, and wound contraction [12].

*Himatanthus bracteatus* (A. DC.) Woodson (*H. bracteatus*) is a species popularly used as a pro-healing natural product [13]. Previous research reports a variety of biological properties of extracts of several species of the *Himatanthus* genus that can contribute to improve wound healing, such as antioxidant and anti-inflammatory [14], analgesic [15], and antimicrobial [16], suggesting a biomedical potential for this plant. Although studies on the chemical composition of extracts derived from *H. bracteatus* are still seldomly reported, iridoids have previously been identified in extracts of the leaves and husk of this plant [17]. These phytochemicals present interesting pharmacological activities, including antioxidant, anti-inflammatory, and antibacterial properties [18], suggesting that it is potentially applicable for healing purposes.

There is a variety of studies focusing on the development of formulations incorporating natural products that work as a system for releasing active substances at the site of the skin injury to accelerate wound healing, including interactive dressings, foams, and biomembranes [19,20,21]. To properly improve wound healing, those ideal formulations should be biocompatible and provide cell attachment, in addition to minimizing the oxidative stress, inflammation, and pain associated with long-lasting wounds [22]. Furthermore, the use of these biomaterials could contribute to reducing wound care-related costs through improvements in healing [23].

The photopolymerizable hydrogel (GelMA) is a biocompatible gelatin-based biopolymer that can be useful as a scaffold for creating complex, cell-responsive microtissues, such as endothelialized microvasculature, or for other applications that require cell-responsive micro-engineered hydrogels [24]. In addition to working as a tridimensional matrix for cell adhesion and migration, GelMA is highly permeable to nutrients, proteins, and oxygen, and can be easily degraded by proteolytic enzymes [25]. Recent studies have demonstrated the successful use of GelMA as a moldable material for filling open wounds that also works to control delivery of substances for wound-healing applications [26,27].

The aim of this work was to evaluate the effect of a novel photopolymerizable gelatin-based hydrogel containing bark-derived ethanol extract of *H. bracteatus* developed for wound-healing applications in a murine model.

## 2. Results

**Figure 1:**
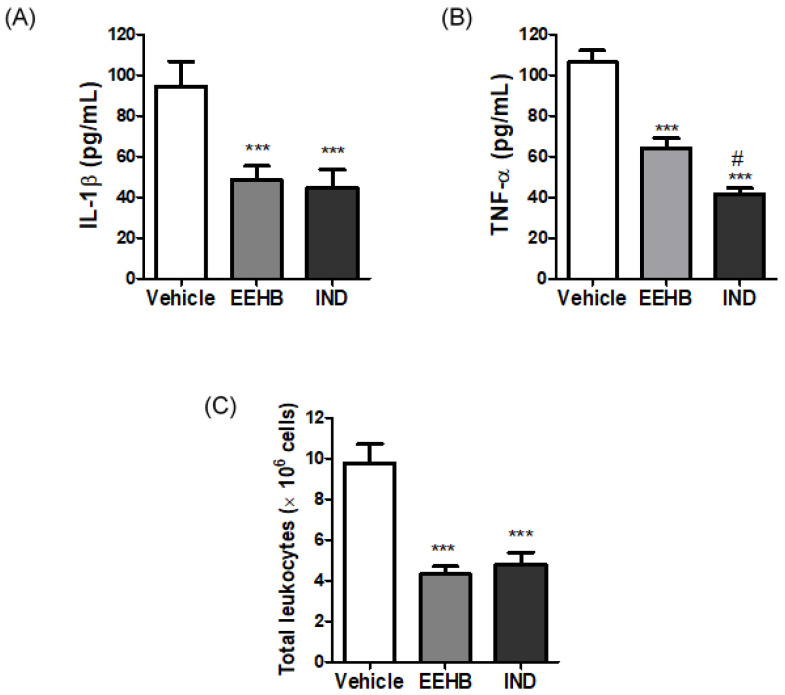
Anti-inflammatory evaluation of EEHB. (**A**) Interleukin-1β (*** *p* < 0.001); (**B**) Tumor Necrosis Factor-α (*** *p* < 0.001 in relation to vehicle group and # *p* < 0.05 in relation to EEHB); (**C**) Total leukocytes (*** *p* < 0.001). All values expressed as mean ± standard deviation. ANOVA followed by Tukey’s test (α = 0.05).

The incorporation of EEHB into GelMA hydrogels was due to anti-inflammatory activity presented by the extract. As demonstrated in Figure 1, the migration of leukocytes to the pleural cavity of the animals was significantly inhibited by the pre-treatment with both EEHB at 10 mg/Kg (4.9 ± 0.1 × 10^6^ cells) and indomethacin (4.8 ± 0.6 × 10^6^ cell) in comparison with the vehicle (9.7 ± 0.9 × 10^6^ cells) (*p* < 0.001). The analysis of the bronchoalveolar lavage fluid showed that the high contents of IL-1β observed in the vehicle (94.4 ± 2.3 pg/mL) was significantly reduced (*p* < 0.001) in EEHB (48.5 ± 6.7 pg/mL) and IND (44.7 ± 8.9 pg/mL), but no significant difference was observed between the two pre-treatments (*p* > 0.05). Similarly, the content of TNF-α obtained in EEHB (64.1 ± 4.9 pg/mL) and IND (41.6 ± 2.8 pg/mL) was significantly lower than in the vehicle (106.3 ± 5.7 pg/mL) (*p* < 0.001). However, the decrease in the TNF-α levels observed in IND was significantly more intense than in EEHB (*p* < 0.05).

**Figure 2:**
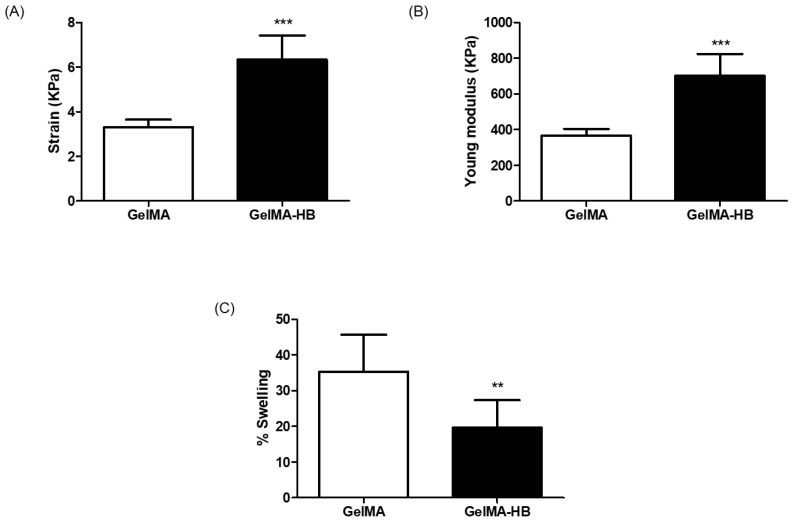
Characterization of GelMA hydrogels. (**A**) Mechanical properties—maximal strain in compression mode (*** *p* < 0.001); (**B**) Mechanical properties—Young modulus in compression mode (*** *p* < 0.001); (**C**) Swelling index (** *p* < 0.01). All values expressed as mean ± standard deviation. Two-tailed *t*-test (α = 0.05).

Concerning characterization of hydrogel formulation, GelMA showed a significantly higher swelling index (*p* < 0.01) when compared to GelMA-HB (Figure 2C). In addition, GelMA-HB was significantly more resistant to compression (*p* < 0.001) when compared to GelMA (Figure 2A,B).

**Figure 3:**
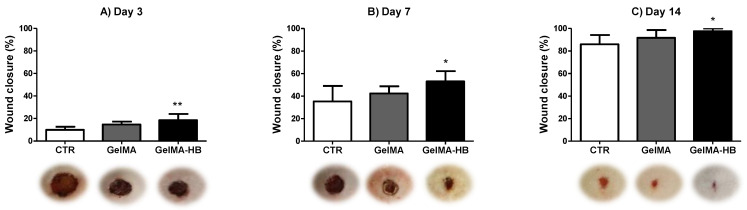
Clinical percentage of wound closure (WCR) over the time course of experimental wound healing on (**A**) day 3, (**B**) day 7 and (**C**) day 14. CTR (untreated control); GelMA (wounds filled with GelMA); GelMA-HB (wound filled with GelMA containing 2% ethanolic extract of *H. bracteatus*). Representative images of wounds from each experimental group are presented at the bottom of the figure. All groups exhibited a significant increase in PWC over time (*p* < 0.001). Significant differences in comparison with CTR group were expressed as: * *p* < 0.05 and ** *p* < 0.01 (two-way ANOVA, followed by Tukey’s multiple comparisons test).

In biological studies, no clinical sign of edema, perilesional hyperemia, or abscess formation was observed over the time course of the experiment, neither in the control group (CTR) nor in the groups treated with GelMA and GelMA-HB, suggesting that the healing course in all the groups was uneventful (Figure 3). All the groups showed a significant and progressive increase in the wound closure rate (WCR) throughout the experimental period, either from 3 to 7 days (*p* < 0.001) or from 7 to 14 days (*p* < 0.001). On day 3, the WCR in the GelMA-HB group (18.5 ± 5.5%) was significantly higher than in the CTR group (9.9 ± 2.7%; *p* < 0.01), but no difference was observed between them and the GelMA group (14.6 ± 1.7%; *p* > 0.05). Similarly, on day 7, the GelMA-HB group (53.0 ± 9.1%) had a WCR significantly higher than the CTR (35.2 ± 13.7%; *p* < 0.05), and once again, no significant difference between them and the GelMA group (42.3 ± 6.3%) was found (*p* > 0.05). On day 14, the average WCR in all the groups was greater than 80%, and although full wound closure was not observed in any animal of theCTR group, it occurred in two animals of both GelMA and GelMA-HB groups. The WCR in the GelMA-HB group (97.6 ± 2.3%) was significantly higher than in the CTR group (85.9 ± 8.2%; *p* < 0.05), but again, similarly to what happened in the previous stages of wound healing, no significant difference between these two last groups and the GelMA group (91.6 ± 6.9%) was observed (*p* > 0.05).

**Figure 4:**
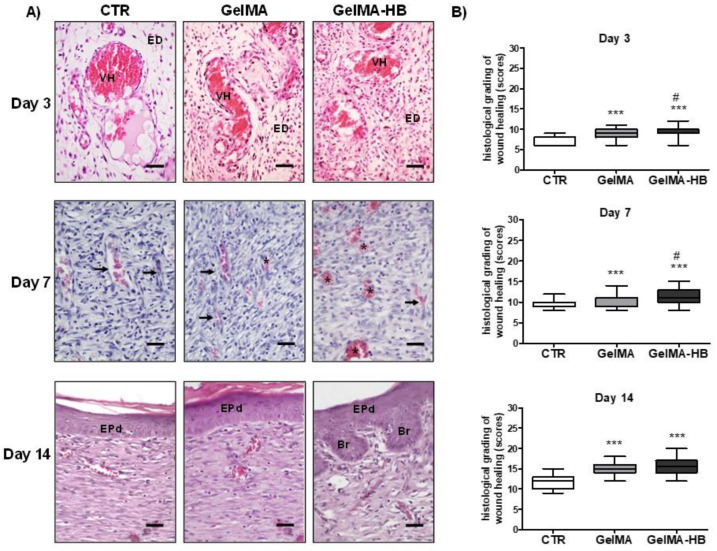
(**A**) Photomicrographs of representative histological sections of the experimental groups. (Day 3) Exudative changes, inflammation, and hyperemic vessels were observed in the three groups. Note less intense connective tissue edema and inflammation on GelMA-HB. (Day 7) Exuberant granulation tissue was observed in the three groups. Note more cellularized tissue and vessels with a more mature morphological appearance in GelMA-HB. (Day 14) Primary fibrous scar and re-epithelialization of the wound surface in the studied groups. The dermo-epidermal interface appears flat in CTR and GelMA, but exhibits epithelial budding (rudimentary cutaneous attachments) in GelMA-HB (HE, 400 x; bar = 20 µm). (**B**) Assessment of histological gradation scores of scar repair over the experimental period (data presented as median and interquartile range). Differences compared to CTR are expressed as *** *p* < 0.001 and differences compared with GelMA are expressed as # *p* < 0.05 (Kruskal–Wallis, followed by Dunn’s multiple comparisons test). Legend: VH—hyperemic vessels; ED—Interstitial edema; arrows—“slit”-shaped capillaries; asterisks—more regular rounded vessels, dilated and congested; EPd—epidermal tissue (stratified squamous epithelium); Br—epithelial buds compatible with rudimentary cutaneous attachments.

Pathological analysis and histological grading of scar repair in the different groups over the time course of wound healing are summarized in Figure 4. Intense inflammatory and exudative changes predominated on day three, exuberant granulation tissue on day seven, and a primary fibrous scar composed of collagenous connective tissue on day 14.

Histological evidence of improved wound healing was observed in GelMA-HB over the whole time course of the experiment, such as: (*i*) mild-to-moderate spindle cell proliferation in the bottom of the wounds on day three; (*ii*) Denser and more cellular granulation tissue on day 7; and (*iii*) full epithelization and epithelial buddings occasionally exhibiting central vorticillary keratinization, compatible with developing cutaneous appendages (rudimentary hair follicles). On day three, the scores of histological grading of wound healing (HGWH) in GelMA-HB (9; 6–12) were significantly higher than in GelMA (8; 6–11; *p* < 0.05) and CTR (6; 6–9; *p* < 0.001). On day seven, GelMA-HB (11; 8–15) also presented significantly higher scores than GelMA (10; 8–14; *p* < 0.05) and CTR (9; 8–12; *p* < 0.001). On day 14, the HGWH in both GelMA (15. 12–18) and GelMA-HB (15; 12–20) groups were significantly higher than in the CTR (12; 10–15; *p* < 0.001). Furthermore, the HGWH in GelMA was significantly greater than in the CTR on days three and seven (*p* < 0.001).

**Figure 5:**
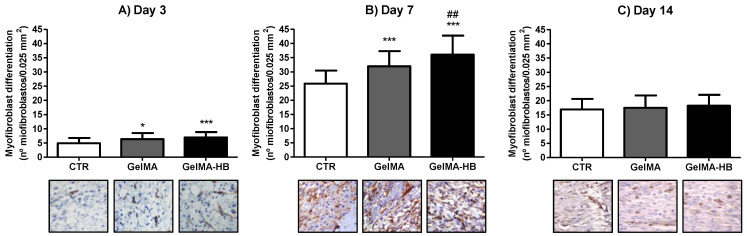
Assessment of myofibroblast differentiation (MFd) over the experimental period (data presented as mean ± standard deviation) on (**A**) day 3, (**B**) day 7 and (**C**) day 14. CTR (untreated control); GelMA (wounds filled with GelMA); GelMA-HB (wounds filled with GelMA containing 2% ethanolic extract of *H. bracteatus*). Significant differences compared to CTR are expressed as * *p* < 0.05 and *** *p* < 0.001; significant differences in relation to GeLMA are expressed as ## *p* < 0.01 (ANOVA, followed by Tukey’s multiple comparisons test). The photomicrographs at the base of the figure constitute histological sections representing the pattern of positive immunostaining for α-SMA in spindle cells in the connective tissue of the scar area in the different groups throughout the experimental period (SABC, 800).

As shown in Figure 5, the lowest MFd indices were seen on day 3. Both in GelMA (6.3 ± 2.1 cells/0.025 mm^2^) and GelMA-HB (6.9 ± 1.8 cells/0.025 mm^2^), the MFd was significantly higher than in the CTR (4.8 ± 1, 9 cells/0.025 mm^2^; *p* < 0.05 and *p* < 0.001, respectively). The peak of mean values occurred on day 7, regardless of the treatment, but the MFd in GelMA-HB (36.1 ± 6.6 cells/0.025 mm^2^) was significantly higher than in GelMA (31.9 ± 5.3 cells/0.025 mm^2^; *p* < 0.01) and the CTR (25.8 ± 4.6 cells/0.025 mm^2^; *p* < 0.001). Furthermore, GelMA showed greater myofibroblast differentiation than the CTR (*p* < 0.001). On day 14, a significant reduction in the MFd was observed in all the groups compared with day 7 (*p* < 0.001). However, there was no difference between the CTR (16.8 ± 3.7 cells/0.025 mm^2^), GelMA (17.5 ± 4.3 cells/0.025 mm_2_), and GelMA-HB (18.2 ± 3.8 cells/0.025 mm^2^) (*p* > 0.05).

**Figure 6:**
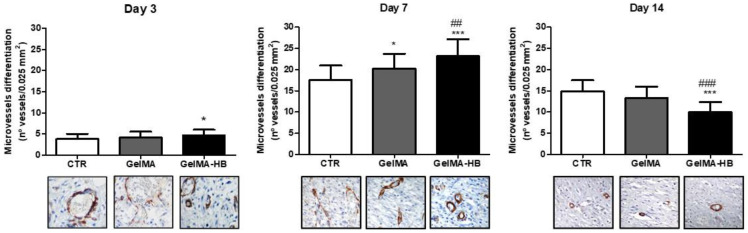
Assessment of microvessel differentiation (MVd) over the experimental period (data presented as mean ± standard deviation). CTR (untreated control); GelMA (wounds filled with GelMA); GelMA-HB (wounds filled with GelMA containing 2% ethanolic extract of *H. bracteatus*). Significant differences compared to CTR are expressed as * *p* < 0.05 and *** *p* < 0.001; significant differences in relation to GeLMA are expressed as ## *p* < 0.01 and ### *p* < 0.001 (ANOVA, followed by Tukey’s multiple comparisons test). The photomicrographs at the base of the figure constitute histological sections representing the pattern of positive immunostaining for CD105 in endothelial cells of capillary blood vessels in the different groups throughout the experimental period (SABC, 800).

The dynamic behavior of the microvessels’ development over the course of wound healing is presented in Figure 6. In all the groups, MVd increased from 3 to 7 days (*p* < 0.001) and then reduced from 7 to 14 days (*p* < 0.01). GelMA-HB presented significantly increased MVd on both days three (4.66 ± 0.2 microvessels/0.025 mm^2^) and seven (23.1 ± 0.7 microvessels/0.025 mm^2^) in comparison with the CTR (3.8 ± 0.2 and 17.5 ± 0.5 microvessels/0.025 mm^2^, respectively (*p* < 0.05). No significant difference in MVd was observed between GelMA-HB and GelMA (4.1 ± 0.3 microvessels/0.025 mm^2^) on day three (*p* > 0.05), but on day seven, GelMA-Hb was significantly greater than GelMA (20.2 ± 0.6 microvessels/0.025 mm^2^) (*p* < 0.01), whereas GelMA was greater than the CTR (*p* < 0.05). In contrast, the MVd in GelMA-HB (9.9 ± 0.4 microvessels/0.025 mm^2^) was significantly lower than in GelMA (13.2 ± 0.5 microvessels/0.025 mm^2^) and the CTR (14.8 ± 0.5 microvessels/0.025 mm^2^) on day 14 (*p* < 0.001), but there was no difference between these last two groups (*p* > 0.05).

**Figure 7:**
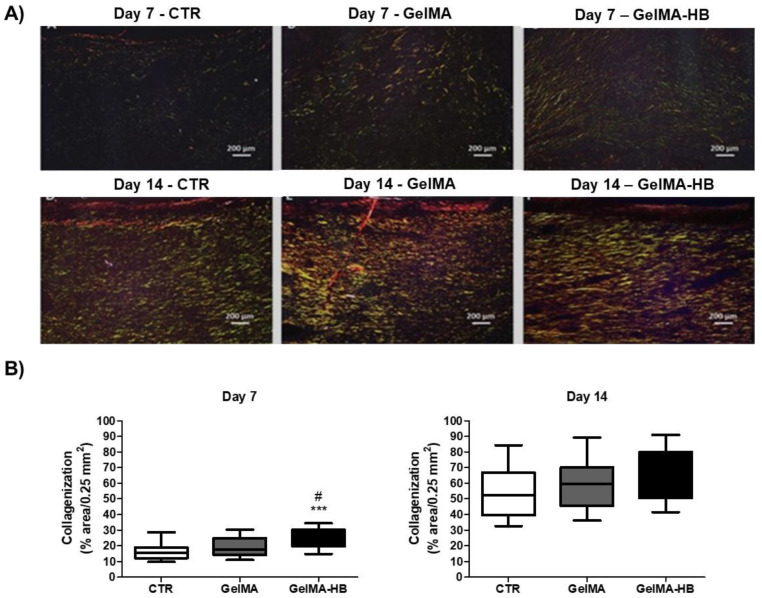
(**A**) Histological sections showing the collagenization pattern in the experimental groups. Few short, thin, delicate collagen fibrils, with reticular arrangement and greenish birefringence (type III collagen) are seen on day 7. Interfibrillar spaces are narrower in GelMA-HB than in the other groups. On day 14, collagen fibers are thicker, longer, and parallel-arranged, with yellow birefringence (type I collagen). (**B**) Histological assessment of the collagenization percentage (%) of the wounds over 7 and 14 days. CTR (untreated control); GelMA (wounds filled with GelMA); GelMA-HB (wounds filled with GelMA containing 2% ethanolic extract of *H. bracteatus*). Significant differences compared to CTR are expressed as *** *p* < 0.001; significant differences in relation to GeLMA are expressed as # *p* < 0.05 (Kruskal–Wallis, followed by Dunn’s multiple comparisons test).

Figure 7 shows the data of the collagenization analysis in the current experiment. On day 3, none of the groups exhibited collagen formation visible under polarized light in the wound area. On day 7, however, all of them showed the formation of a very delicate network of short and thin collagen fibrils, showing a greenish-yellow birefringence pattern compatible with type III collagen. The interfibrillar spaces were wide, irregular, and limited by inter-crossed birefringent fibrils (reticular or “mesh” appearance). The interfibrillar spaces comprising the collagen network were shorter in GelMA-HB than in the other groups. On day 14, the collagen network was composed of thicker and longer collagen fibers showing golden yellow or slightly reddish birefringence, consistent with type I collagen. The fibers were organized in parallel arrangement in relation to the wound surface, with tiny, irregular, and narrow interfibrillar spaces. The collagenization density was very similar between the groups. The quantitative analysis of collagenization on day 7 showed significantly higher percentages of collagen deposition in GelMA-HB (22.6%; 19.7–30.3%) than in the CTR (15.6%; 12.3–18.9%; *p* < 0.001) and GelMA (17.6%; 14.0–24.9%; *p* < 0.05). On day 14, the percentage of collagen deposition increased significantly in all the groups (*p* < 0.001), but there was no significant difference (*p* > 0.05) between the CTR (52.3%; 39.4–66.8%), GelMA (59.6%; 36.2–70.1%), and GelMA-HB (58.7%; 50.6–79.9%).

## 3. Discussion

In this study, the choice of GelMA as a biomaterial for in situ delivery of the extract for wound-healing applications was based on the analysis of its physical and biological properties. Gelatin has many excellent characteristics such as excellent biocompatibility, biodegradability, nonimmunogenicity, and inducing cell adhesion and proliferation [25]. The gelatin hydrogel, in turn, is a cross-linked polymeric mesh formed by hydrophilic chemical groups that facilitate the absorption of water, and which shows similarity to the extracellular matrix [28]. However, hydrogels often exhibit poor mechanical characteristics, which makes their use in certain applications difficult [29]. Hence, the methacrylic anhydride and a photoinitiator agent (Igarcure) are added to the gelatin polymer to form GelMA, a modified polymer that undergoes rapid formation of chemical bonds between the amino and anhydride groups after exposure to UV light (photoinduced polymerization), reducing viscosity and increasing the mechanical strength of the biomaterial [24,30,31]. GelMA has high permeability of nutrients, proteins, and oxygen, and proteolytic degradability [24,32], in addition to providing an adequate framework for proper cell adhesion and favoring vascular morphogenesis [33].

In this study, we added EEHB before the hydrogel formation. After photopolymerization, the tridimensional formulation with EEHB showed less swelling and increased resistance to mechanical compression. This can be justified as iridoids existing in this extract having hydrogen ions from the hydroxyls and oxygen belonging to the furan ring associated with the production of reactive species during the photopolymerization reaction, which can form bonds altering the degree of crosslinking [34].

Clinical analysis of the WCR is considered an essential tool to analyze the speed and quality of the healing process, as it provides data to suggest improvement or worsening of wound healing [33,35]. In this study, GelMA-HB, but not GelMA alone, accelerated wound closure over the entire experimental time, suggesting that the chemical constituents present in EEHB, such as iridoids, plumericine, and isoplumericine, may play a biomodulatory role in different stages of wound repair. In the early phases of wound healing (acute inflammatory phase), the more intense the acute inflammation, and, consequently, the release in situ of vasoactive chemical mediators (e.g., prostaglandins and nitric oxide), the more intense the interstitial edema [36], which tends to increase the wound diameter. As EEHB demonstrated an anti-inflammatory effect, the smaller mean wound area observed in GELMA-HB could have occurred due to the lower edematous distension of the perilesional tissues. Furthermore, plumericine, one of the major chemical markers of EEHB, has previously proved to reduce pro-inflammatory mediators, such as tumor necrosis factor-α (TNF-α), cyclooxygenase-2 (COX-2), and inducible nitric oxide synthase (iNOS), supporting the theory of an important anti-inflammatory effect played by the extract in the early stages of wound healing. In later stages, wound closure has been directly related to transdifferentiation of fibroblasts into myofibroblasts, with consequent contraction of the lesion margins in a centripetal direction. Myofibroblasts begin to differentiate in the wound area from the third day after the initial injury [37], which justifies the low wound retraction rates on day 3. The peak of myofibroblast differentiation, in a murine model of second intention wound healing, occurs around day 7, at which point there is a progressive reduction in this cell population [38], as also observed in the present study. As GelMA-HB presented a significantly higher WCR over the course of wound healing until day 14, the hypothesis that the phytochemical components found in EEHB could have accelerated or intensified myofibroblast differentiation in vivo was brought up and analyzed.

Histopathological analysis of wound biopsies can bring interesting insights into healing dynamics [39], but to provide significant information on the healing progress it must properly include the basic components of the healing process, such as angiogenesis, inflammation, fibroplasia and connective tissue matrix restoration, wound contraction and remodeling, and epithelialization [40]. The scoring system used in the current work was designed in such a way that lower scores are associated with unhealed wounds, whereas higher scores are associated with completely closed and healed wounds without excessive scarring. The histological features of wound healing observed in GelMA-HB, represented by the reduction of the inflammation, earlier maturation of the granulation tissue, and better epithelization and scarring, in addition to higher scores of histological grading, are suggestive of improvement in wound healing. However, the good performance of GelMA-HB cannot be exclusively attributed to the anti-inflammatory effects of the EEHB previously demonstrated in the current study, as a range of improvements in different phases of wound healing were observed. These data support the hypothesis that the biomaterial could act in other stages of healing, such as myofibroblast differentiation or the formation of granulation tissue.

For the determination of MFd, only immunomarked cells dispersed in the connective tissue, following or not following the orientation of collagen fibers and fibrils, were considered in the counts. Immunomarked cells surrounding the blood vessel wall were interpreted as pericytes and excluded. Myofibroblasts are contractile fibroblasts derived from a wide variety of histogenetic sources, such as: (*i*) dermal or mucosal fibroblasts; (*ii*) smooth muscle cells; (*iii*) perisinusoidal cells; (*iv*) epithelial cells, such as keratinocytes (epithelial-mesenchymal transition) and endothelium (endothelial-mesenchymal transition); and (*v*) circulating totipotent mesenchymal cells (stem cells) [41]. Myofibroblasts are often characterized by the cytoplasmic expression of α-SMA [42]. The role of TGF-β in the differentiation of fibroblasts into myofibroblasts is well-established. However, lack of expression of actin filaments and drastic collapse of the fibroblast cytoplasm, a completely opposite phenotype to myofibroblasts with ample and bulky cytoplasm rich in contractile filaments, has been reported in response to increased levels of PGE-2 in tissues previously treated with TGF-β [43]. Thus, the low myofibroblast differentiation on day 3 might have resulted in the high content of prostaglandins, including prostaglandin E2 (PGE2), during the inflammatory phase of wound healing, particularly secreted by polymorphonuclear neutrophils and M1 macrophages. The higher MFd found in GelMA and GelMA-HB probably occurred because the application of a collagen-based moldable filling material not only reduces the natural influx of macrophages, as long as it significantly reduces the need to remove debris and hemostatic material, but also promotes a fast and dynamic interaction between the cells and the ECM, leading to rapid resolution of inflammation and increased growth and differentiation of fibroblasts [35]. The subsequent increase in MFd is likely a response of the replacement of M1 macrophages (sources of pro-inflammatory cytokines, such as PGE-2) by M2 macrophages (sources of TGF-β) [44]. The significant increase in MFd in GelMA-HB compared with GelMA on day 7 seems to be the first evidence that the phytochemical constituents of EEHB (plumericin or isoplumericin) could act in myofibroblastic differentiation in vivo. On the other hand, the possibility that this over-differentiation of myofibroblasts is not the result of a direct action of the extract components, but a secondary effect of the anti-inflammatory activity, anticipating the transition of M1 to M2 macrophages, promoting increased release of TGF-β, and consequent earlier myofibroblastic differentiation, cannot be ruled out. However, further studies are needed to determine the precise mechanisms underlying the increase in MFd. On the other hand, the persistence of myofibroblasts in a closed wound is strongly suggestive of the development of a hypertrophic scar, a pathological evolution of wound healing [45]. This phenomenon is based on the fact that, in addition to type I and type III collagen, myofibroblasts also produce a myriad of other constituents during wound repair and fibrosis, such as collagen types IV, V, and VI, glycoproteins, and proteoglycans, such as fibronectin, laminin, and tenascin [46]. Therefore, the progressive reduction of MFd in GelMA-HB to values comparable to the other groups is suggestive that this biomaterial is not supposed to induce excessive fibrosis at the end of the experimental time.

Microvasculature development is critical for wound healing success, as it maintains the oxygen and nutrient supply to the injured area, allowing cell proliferation and collagenization [47]. In this study, newly formed blood vessels were identified by the cytoplasmic positivity of endothelial cells for the endoglin antigen (CD105), a transmembrane protein component of the transforming growth factor receptor-β (TGFβR), which is expressed on vascular endothelial cells in all stages of maturation [48]. The increase in the content of capillary vessels from day 3 to day 7 was expected because approximately four days after tissue injury, there was an increase in the population of type 2 macrophages at the injury site, which are the source of the main growth factors responsible for endothelial proliferation, such as VEGF and FGF-2 [6]. Subsequently, there was significant reduction in the vascular content in all groups, likely due to two major biological mechanisms: (*i*) negative feedback on in situ production of VEGF promoted by the increase in tissue oxygen supply, inhibiting endothelial proliferation [49]; and (*ii*) release of several negative angiogenesis regulators in the resolving wounds, including IP-10 (CXCL10) [50] and Sprouty2 [51]. Recently, a possible role of antioxidant compounds in TRPM2 (Transient receptor potential melastatin 2)-mediated Ca^2+^ signaling VEGF-induced angiogenesis has been suggested [52].

In addition, antioxidant chemical compounds, such as plumericin, prevent endothelial cell senescence by blocking the activation of the IKK2 enzyme, a protein kinase responsible for suppressing inhibitors of the NF-κB pathway. This process results in a reduction of TNF-α-induced ROS production to basal levels, so that this biological activity is closely associated with the antioxidant potential of this iridioide [53].

On the other hand, GelMA-induced MVd increased on day seven, possibly because drug delivery systems that mimic the extracellular matrix (ECM), such as gelatin-based biomaterials, provide a morphostructural microenvironment that guides vessel formation and acts as a scaffold or framework for angiogenesis and further tissue regeneration [54]. Additionally, the unusual intermolecular crosslinking of collagenous matrices, as occurs in GelMA, seems to work as a fibrillar microstructure adjustment process, forming a semi-rigid 3D framework that provides better proliferation and migration of endothelial colonies (ECFC) to form three-dimensional lumenized vascular networks in vitro [55]. Hence, those physicochemical and biological phenomena could justify the results obtained by GelMA. Among the three groups, GelMA-HB exhibited the greatest reduction in MVd on day 14. This finding suggests better wound healing, since the reduction of the vascular content also seems to play an important role in fibrous scar formation. In fact, uncontrolled vessel growth or impaired vessel regression resulting from an excessive inflammatory response can result in dysfunctional scarring [56]. Furthermore, wounds that heal exceptionally well—such as fetal skin and oral mucosa wounds—have much less angiogenesis than adult skin wounds, which normally form more visible and dysfunctional scars [5]. These data suggest that wounds that exhibited less intense inflammation, such as those in GelMA-HB, would tend to present a more rapidly maturing capillary network, and thus less fibrous scar formation, a morphological hallmark of a better repair process.

The histochemical analysis of the collagenization was performed to assess the effects of the biomaterials (GelMA and GelMA-HB) on the pattern of collagen deposition over the wound healing time course, using polarized light microscopy. Higher amounts of type III collagen fibrils observed in GelMA-HB on day 7, as in morphological and morphometric analysis, might have contributed to creating a denser and more regular collagen network in the healing wounds, favoring the attachment and migration of endothelial cells to form new capillary blood vessels. These data support our previous findings of more rapid formation and maturation of granulation tissue in this group. However, the amounts of type I collagen were not enhanced on day 14, even in GelMA-HB. These data suggest that the earlier increase in type III collagen does not necessarily promote a later increase in type I collagen. Moreover, increased myofibroblast differentiation and scar deposition, as well as impaired wound closure, have been demonstrated in type III collagen-deficient mice [57].

## 4. Material and Methods

### 4.1. Ethanolic Extract of H. bracteatus (EEHB)

Ethanolic extract of *H. bracteatus* (EEHB) was previously prepared and characterized according to the methodology described in the patent BR 10 2017 007,641 5 (2016). Briefly, the ethanolic extract was obtained from bark of *Himatanthus bracteatus* (10 g powder through 18 to 32 mesh) using a pressure extraction unit (100 bar) and a sequence of hexane, dichloromethane, ethyl acetate, and ethanol as solvents (250 mL each solvent) at 40 °C during 20 min for each solvent. The flux of extractor solvent was 2 mL/min. This natural extract was granted by the inventors. EEHB presented iridoids such as 15-desmethylplumeride, 13-deoxyplumeride, formic acid, isoplumeride, and plumeride.

### 4.2. Preparation and Characterization of Hydrogels

#### 4.2.1. Pre-Polymer Preparation

To obtain the gelatin methacryloyl (GelMA) pre-polymer, firstly 10% (*w*/*v*) gelatin was dissolved into Dulbecco’s phosphate buffered saline (DPBS; GIBCO) at 60 °C, pH 7.4, and methacrylic anhydride (1% *v*/*v*) was added and allowed to react for 3 h at 50 °C. Following this, the mixture was dialyzed against distilled water for 1 week, followed by a lyophilization process using a Liotop L101 (Liobras, São Carlos, São Paulo, Brazil) at −55 °C and vacuum pump at 0.03–0.2 mbar for 5 consecutive days.

#### 4.2.2. Hydrogel Formulations (Photopolymerization Step)

GelMA prepolymer solution (10 *w*/*v*%) was mixed with the photoinitiator (2-hydroxyl-1-[4-(2-hydroxyethoxy) phenyl] -2-methyl-1-propanone, Irgacure 2959) (0.5 *w*/*v*%, Sigma-Aldrich, St. Louis, MO, USA) in DPBS at 80 °C. Photocrosslinking was achieved by exposing the solution to 6.7 mW cm^−2^ UV light Philips TL40W/12 RS (light intensity of 8.6 × 10^4^/cm^2^) (Koninklijke Philips N.V., Amsterdam, The Netherlands) for 60 s. The same formulation was prepared adding 2.0% EEHB to the solution to obtain the GelMA-HB (test material).

#### 4.2.3. Characterization of Hydrogels

At first, cylindrical samples of light-cured GelMA or GelMA-HB were molded using 250 µL of solution in a circular mold (*n* = 8, 0.75 mm in diameter) and photopolymerized as previously described. (*i*) Mechanical test: Samples were submerged into 300 μL of PBS solution and incubated at 37 °C for 24 h. Each sample was dried lightly using a napkin and tested in a compressive mode at a rate of 2 mm/s on a TA-TX2 texturometer (Stable Micro Systems, Godalming, England). The compressive modulus was determined as the slope of the linear region corresponding with 0 and 5% strain [24]. (*ii*) swelling analysis: Samples were placed in 300 μL of PBS at 37 °C for 24 h. Samples were removed, dried lightly using a napkin to remove the residual liquid, and the swollen weight was recorded. Samples were then lyophilized and weighed once more to determine the dry weight of the polymer. The mass swelling ratio was then calculated as the ratio of swollen hydrogel mass to the mass of dry polymer and expressed in percentage.

### 4.3. Biological Assays

#### 4.3.1. Ethics Issues

The ethics issues were addressed according to the National Animal Experiment Control Council for experiments in animals and applied in this study, which was approved by the Ethical Committee for Animal Experimentation (approval nº 011114).

#### 4.3.2. Anti-Inflammatory Activity of EEHB

Thirty male Wistar rats (280 ± 20 g, 8-weeks-old) were assigned into five groups and pretreated with freshly prepared samples by oral administration (0.1 mL/10 g) of vehicle (saline), EEHB (10 mg/kg), and indomethacin (10 mg/kg). After 1 h, carrageenan (300 mg/0.1 mL saline) was administered by intrathoracic (i.t.) injection to induce pleurisy, and four hours later, the animals were euthanized using a lethal dose of ketamin and Xylazin (1:1, 20 mg/kg). Pleural cavities were washed with 1 mL PBS (1x) containing EDTA (10 mM) and then immediately aspirated. Samples were centrifuged (770× *g* for 10 min), supernatant was stored at −80 °C, and the pellet was resuspended in 250 μL of PBS. The total number of leukocytes was assessed by direct counting in a hemocytometer using the Trypan blue exclusion method. For cell differentiation analysis, 200 μL of cell suspension was added in a cytospin slide chamber (Shandon EZ Double Cytofunnel, Thermo Scientific, Waltham, MA, USA), spun at 800 rpm for 5 min in a Cytospin 4 (Thermo Fisher Scientific, Waltham, MA, USA), and stained with Stain Set Diff-Quik (Siemens Healthcare Diagnosis Inc., Newark, NJ, USA). Percentages of macrophages, neutrophils, and lymphocytes were obtained and adjusted by total cell number. In addition, TNF-α and IL-1β contents were assessed in previously centrifuged (770× *g* for 10 min) samples of lung lavages from animals treated with EEHB and indomethacin (both at 10 mg/kg), using ELISA according to the manufacturer’s protocol (BD-Bioscience Pharmingen, San Diego, CA, USA). Data were expressed as pg/mL [58,59].

#### 4.3.3. Wound-Healing Assay GelMA Formulations

A total of 54 male Wistar rats (*Rattus norvegicus albinus*, 300 ± 20 g) were kept in individual polypropylene cages (33 × 17 × 40 cm) in a controlled environment (25 ± 2 °C, 50 ± 5% relative humidity, 12 h light/dark cycle, with water and feed ad libitum). The animals were anesthetized with intraperitoneal administration of xylazine/ketamine (1:1, 5 mg/kg), and their backs were shaved. Antisepsis was performed using topical 1% povidone-iodine. Full-thickness rounded wounds were carried out on the back of each animal (1 cm under the interscapular area) using an 8 mm diameter biopsy punch (Dermatological punch #8, Rhosse^®^). Subsequently, the animals were randomly assigned into three groups (*n* = 18): CTR (control group, wounds filled with topical application of 40 µL of saline), GelMA (wounds filled with 40 µL of gelatin-based photopolymerizable hydrogel), and GelMA-HB (wounds filled with 40 µL of gelatin-based photopolymerizable hydrogel containing 2% EEHB). The wound-filling materials (GelMA and GelMA-HB groups) were subjected to UVB light exposure, as previously described in this paper. The saline group was subjected to simulated irradiation for 60 s. After the end of the surgery, the animals received potassium diclofenac (10 mg/kg, i.m.) to minimize the postoperative symptoms. After 3, 7, and 14 days, six animals from each group were euthanized using a lethal dose of xylazine/ketamine (1:1, 20 mg/kg). After death certification, the injured areas were surgically removed (approximately 1.0 cm of margins) and fixed in a 10% formalin solution (pH 7.4) 48 h.

#### 4.3.4. Assessment of Wound Closure Rates (WCR)

On days 0 (immediately after surgery), 3, 7, and 14 after surgery, the wounds were photographed using a digital camera (Cybershot Sony HX-300) fixed on a tripod with a standardized height of 20 cm from the wound surface. The images were analyzed using the software Image J^®^ (National Institutes of Health, Bethesda, MD, USA) to obtain a value for the total wound area. The percentage of wound contraction was calculated using the following equation:

$$WCR=\frac{WA_{i}-WA_{f}}{WA_{i}}\times 100$$

where WCR stands for the wound closure rate, WA_i_ for the initial wound area (day 0), and WA_f_ for the final wound area (days 3, 7, or 14). Daily clinical evaluation of the wounds was also performed, according to the presence of clinical signs of atypical course of wound healing, such as edema, suppuration, and hyperemia.

#### 4.3.5. Pathological Analysis of the Healing Tissues

Formalin-fixed specimens were dehydrated, cleared, and embedded in paraffin, according to routine histological processing techniques. A total of 18 serial histological sections (5 µm thick) were obtained from each embedded specimen, three of them (sections 1, 7, and 13) stained in HE (routine staining) and three in Sirius red (sections 4, 10 and 16). HE-stained histological sections were used to perform a descriptive analytical study of the inflammatory infiltrate, granulation tissue, primary fibrous scar (dense connective tissue formed because of fibrosis of the granulation tissue), and epithelization process.

i. Histological grading of wound healing (HGWH): To perform the histological grading of wound healing, a semiquantitative scoring system based on an ordinal scale was used, considering six histological criteria related to the healing process [60], as shown in Table 1.

**Figure 8:**
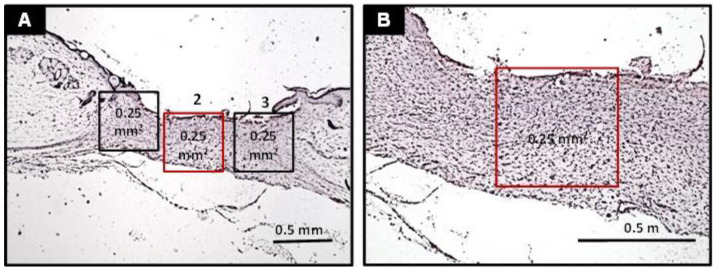
(**A**) Strategy of histological fields selection at low-power magnification for further histological analysis (40×). (**B**) Photomicrograph of histological field 2 (in red) at medium magnification, showing the proper delimitation of the area (analytical area of 0.25 mm^2^) for further histological analysis (100×). The same procedure was also performed in areas 1 and 3 and repeated in all histological sections.

Three histological fields obtained from photomicrographs at 100× magnification (analytical optical area of 0.25 mm^2^) in each histological section were selected and analyzed (one from each edge and one from the center of the wounds—Figure 8). The determination of the final healing score in each case was obtained by adding the scores of the individual criteria. The data obtained were expressed as median, interquartile range, and maximum and minimum values. All histological sections were examined by two examiners, who were blinded to the groups during all the histological analysis performed in this study.

ii. Immunohistochemical analysis of myofibroblast differentiation (MFd) and microvascular density (MVd): Histological sections were mounted on glass slides previously salinized, deparaffinized in xylol, and washed in decreasing concentrations of ethyl alcohol (100%, 95%, 90%, 80%, and 70%). Blocking of endogenous peroxidase activity was performed with 3% hydrogen peroxide and methyl alcohol (10 min in a dark room). Then, the procedure of immunodetection of the researched antigens was carried out by means of incubation with the primary antibodies, as described in Table 2.

Prior to incubation of both antibodies, antigen recovery was performed by moist heat under pressure in 10 mM citrate buffer/pH 6.0 solution. For MFd immunodetection, histological sections 2, 5, 8, 11, 14, and 17 were used, whereas for MVd immunostaining, sections 3, 6, 9, 12, 15, and 18 were used. The histological sections were incubated with a secondary antibody (SABC—Streptavidin Biotin Complex, catalog number SA1022) at 37 °C for 30 min. The revelation of the reaction was carried out by incubating the sections with chromogene diaminobenzidine (DAB, Ventana Medical Systems, Tucson, AZ, USA) in a darkroom for 30 min. Counterstaining was performed with Meyer’s hematoxylin. Both steps were performed with intervals of four minutes each. Two histological sections of previously diagnosed oral myofibroma were used as a positive control for antibody 1A4, while two sections of pyogenic oral granuloma were used as a positive control for the CD 105 antibody. The negative control was obtained using two other sections from the same cases, replacing the incubation of the primary antibody by TRIS-HCl. The number of α-SMA-positive cells and small CD105-positive blood capillaries were counted in ten fields histological series of each histological section (400×, analytical area corresponding to 0.025 mm^2^, 6 sections/case). Final data were expressed as mean ± standard error medium (SEM) cells/0.025 mm^2^.

iii. Assessment of collagen fiber content: the analysis of collagen fibers was carried out in three histological sections of each animal stained in Sirius red and analyzed under polarized light. Collagen fibers were classified into type III or type I according to their birefringence pattern (green and yellow/red, respectively). The morphological features (stretched/wavy, thin/thick, short/long) and architectural arrangement (reticular, parallel or fascicle) of the fibers were also observed. Therefore, three histological fields (100× magnification, 0.25 mm^2^) of each histological section were photomicrographed, and the percentage of the area containing collagen fibers was obtained using the software Image J^®^.

### 4.4. Statistical Analysis

The PRISM 7.0 software (GraphPad Software: La Jolla, CA, USA) was used to carry out the statistical analysis. All the data sets were subjected to analysis of normality distribution using the Shapiro–Wilk test. Data sets with symmetric (Gaussian) distribution were expressed as mean ± standard deviation (SD), and differences between means were analyzed using the analysis of variance test (two-way ANOVA) followed by Tukey’s multiple comparisons test or two-tailed *t*-test (for two-sample analysis). Data sets with asymmetric (non-Gaussian) distribution were expressed as median and interquartile range, and differences between means were analyzed using the Kruskal–Wallis test followed by Dunn’s multiple comparisons test. A significance level of 5% was adopted in all statistical tests applied in this study.

## 5. Conclusions

GelMA-HB improved wound healing by accelerating wound closure and improving histological healing grading, collagenization, and myofibroblastic and microvascular differentiation in rodents. However, further studies are still needed to determine whether such activity resulted from the direct action of phytoconstituents on fibroblasts and endothelial cells, or just a secondary effect of the anti-inflammatory activity of iridoids in earlier phases of wound healing.

## Figures and Tables

**Table 1:** Parameters assessed to calculate wound healing score.

Score System	Histological Criteria	Histological Staining and Analytical Method
plenty—1, moderate—2, a few—3	Inflammatory infiltrate	Light microscopy (HE)
profound—1, moderate—2, scanty—3, absent—4	Amount of granulation tissue	Light microscopy (HE)
vertical—1, mixed—2, horizontal—3	Orientation of collagen fibers	Polarized light (sirius red)
reticular—1, mixed—2, fascicle—3	Pattern of collagenization	Polarized light (sirius red)
profound—1, moderate—2, minimum—3, absent—4	Amount of early collagen (type III)	Polarized light (sirius red)
profound—1, moderate—2, minimum—3	Amount of mature collagen (type I)	Polarized light (sirius red)

**Table 2:** Target antigens and antibodies used in the immunohistochemical analysis of myofibroblast differentiation (MFd) and microvascular density (MVd).

Antigen	Target Cell	Clone	Dilution	Incubation
α-SMA	Myofibroblasts	1A4 (Dako)	1:100	30 min
CD105	Endothelial cells	SN6H (Dako)	1:500	Overnight (18 h)

## Data Availability

Not applicable.
